# L'encéphalomyélite aiguë disséminée chez l'enfant

**DOI:** 10.11604/pamj.2014.19.280.4720

**Published:** 2014-11-14

**Authors:** Ilham Tadmori, Sana Chaouki, Sana Abourazzak, Souilmi Fatima Zahra, Sarra Benmiloud, Mounia Lakhdar Idrissi, Samir Atmani, Moustapha Hida

**Affiliations:** 1 Service Pédiatrie, Département mère-enfant, CHU Hassan II, Fès, Maroc

**Keywords:** Enfant, ADEM, IRM, corticoïdes, child, acute disseminated encephalomyelitis, MRI, corticosteroids

## Abstract

L'encéphalomyélite aiguë disséminée (ADEM) est une maladie inflammatoire, démyélinisante, multifocale intéressant principalement la substance blanche du système nerveux central. Elle est rare mais non exceptionnelle chez l'enfant. Les auteurs rapportent une étude colligeant 9 cas d'ADEM pris en charge au service de Pédiatrie du CHU Hassan II à Fès, sur une période de 4 ans. Il s'agit de cinq garçons et quatre filles; âgés entre 2 ans et 13 ans. Les antécédents (ATCD) d'infection virale sont notés chez quatre patients. La fièvre est constante. Les convulsions sont présentes dans cinq cas et les troubles de conscience dans cinq cas. L'examen neurologique était anormal. L’étude de liquide céphalo-rachidien a révélé une réaction méningée. On ne notait pas de syndrome inflammatoire biologique. L'imagerie par résonance magnétique (IRM) cérébrale a objectivé des lésions en hyper-signal en T2 et T2 FLAIR chez tous les patients. Pour le traitement, des bolus de corticoïdes intraveineux ont été administrés, relayés par une corticothérapie orale et associés à une kinésithérapie motrice et un traitement antiépileptique pour certains patients. L’évolution de nos malades était favorable globalement et on a déploré le décès d'une patiente. L'ADEM est une affection rare du système nerveux central. Les critères diagnostiques sont fondés sur des éléments cliniques et des données d'imagerie. La prise en charge doit être précoce et multidisciplinaire.

## Introduction

L'encéphalomyélite aigue disséminée (ADEM: acute disseminated encephalomyelitis), appelée également encéphalite post-infectieuse, encéphalite post vaccinale ou encéphalite péri-veineuse, est une affection inflammatoire démyélinisante multifocale du système nerveux central. Le mécanisme est auto-immun suite à une vaccination ou un épisode infectieux le plus souvent viral. Le tableau clinique associe des troubles de la conscience, différents signes de déficit neurologique et des anomalies de la substance blanche à l'IRM [1]. Elle est plus fréquente chez l'enfant que chez l'adulte. La prise en charge est basée sur la corticothérapie intraveineuse par de méthyl-prednisolone à hautes doses.

## Méthodes

Notre travail est une étude rétrospective de neuf cas d'ADEM colligés au sein du service de pédiatrie au CHU Hassan II de Fès sur une période de 4 ans allant du janvier 2010 au Décembre 2013. L’étude a porté sur tous les cas d'ADEM ayant été hospitalisés et pris en charge au service. Le but de travail est d'analyser les aspects cliniques, radiologiques, thérapeutiques et évolutifs de cette pathologie.

## Résultats

L´âge de nos patients est compris entre 24 mois et 13 ans, avec une moyenne d´âge de 4 ans et demi. La tranche d’âge la plus touchée est celle de 2 - 6 ans (78%) et le sex-ratio est de 1,25. Une infection virale prodromique a été notée chez quatre patients. Une varicelle précédant les symptômes neurologiques a été révélée dans deux cas, des oreillons dans un cas, une fièvre éruptive dans un cas. Le délai entre l'infection virale et le début de la symptomatologie varie entre six jours et 60 jours. Aucun de nos patients n'a reçu de vaccin dans le mois précédant l’épisode. Tous nos patients sont admis pour association de signes généraux: fièvre, vomissement, céphalées... et de manifestations neurologiques: trouble de conscience, signes neurologiques focaux hémisphériques ou médullaires.

**Figure 1:**
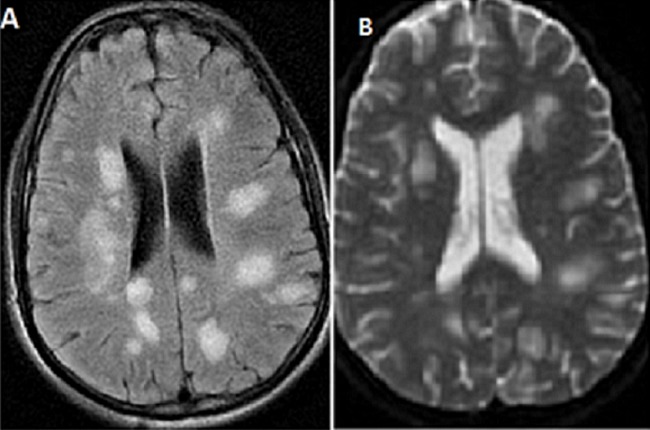
(A): IRM cérébrale séquence T2 FLAIR; (B): T2 montrant des lésions multifocales en hyper-signal de la substance blanche

**Figure 2:**
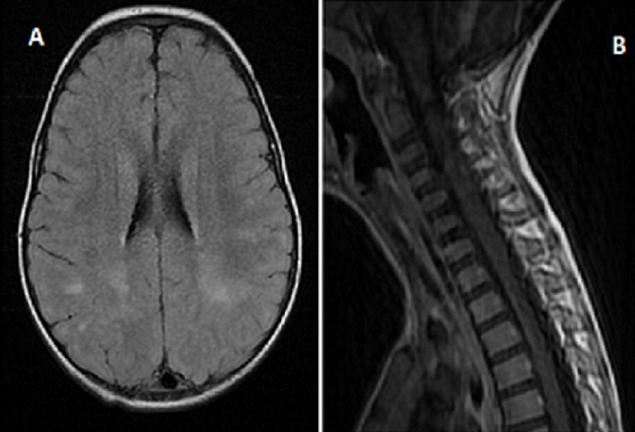
(A, B): IRM cérébro-médullaire séquence T2 FLAIR; lésions multifocales en hyper-signal

**Figure 3:**
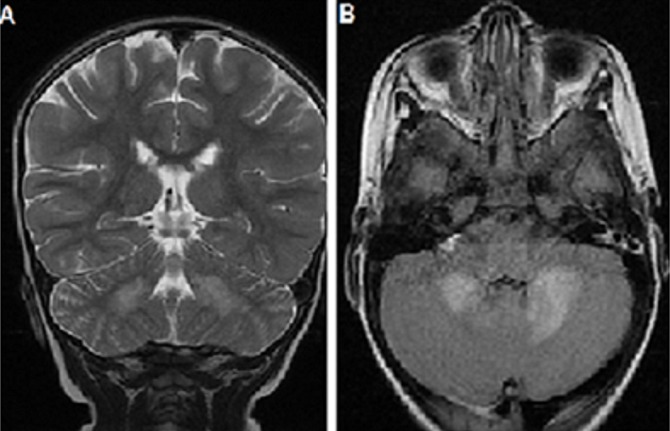
(A): IRM cérébrale séquence T2 axiale; (B): T2 FLAIR axiale montrant des hyper-signaux cérébelleux et de la substance blanche sus-tensoriel

Le délai entre le début de la symptomatologie et l'admission dans notre service est variable de un jour à vingt-cinq jours avec une moyenne de sept jours. La fièvre est constatée dans tous les cas et les convulsions dans cinq cas soit 55,5% des cas. Les crises sont de type tonico-cloniques généralisées dans trois cas et hémi-corporelles dans deux cas. Les troubles de conscience avec des GCS entre 8 et 13 sont observés dans cinq cas. Les troubles de la marche dans sept cas. Pour tous les cas, l'examen neurologique est anormal: un syndrome cérébelleux est objectivé dans six cas (66%). Une hémiparésie est retrouvée dans trois cas (33%), des signes d'irritation pyramidale dans deux cas (22%) et une paraplégie dans un cas soit 11% des cas. La ponction lombaire a révélé une réaction méningée chez huit patients avec des leucocytes entre 10 éléments à 87 éléments. Le bilan inflammatoire est négatif sauf pour un patient qui a présenté une infection respiratoire associée. La tomodensitométrie a montré des lésions hypo-denses dans trois cas, un œdème cérébral dans un cas. L'imagerie par résonnance magnétique cérébrale a été anomale chez tous les patients objectivant des lésions de la substance blanche en hypo-signal T1 et en hyper-signal T2 et T2 FLAIR, associées à une atteinte de la substance grise chez quatre patients. L'atteinte médullaire est constatée dans un cas (Figure 1, Figure 2, Figure 3).

Le traitement chez nos patients est basé sur des bolus de corticoïdes intraveineux administrés 3 jours de suite, relayés par une corticothérapie orale à dose de 2mg/kg/j pendant une durée variant de 4 à 6 semaines selon la sévérité de tableau clinique. Une kinésithérapie motrice est indiquée pour nos patients. L'antibiothérapie parentérale ou orale est administrée dans 55,5% des cas. L'acyclovir est administré chez 44,5% des patients à l'admission et il est arrêté après les résultats d'imagerie. Un traitement antiépileptique est utilisé pour 44,5% des malades. L’évolution globale de nos malades a été favorable chez 89%. Un patient a présenté une évolution bi-phasique avec une rechute après six mois. Un patient a gardé des séquelles à type d'ataxie et un est décédé.

## Discussion

L'encéphalomyélite aigue disséminée (ADEM: acute disseminated encephalomyelitis), appelée également encéphalite post-infectieuse, encéphalite post vaccinale ou encéphalite péri-veineuse, est une maladie inflammatoire démyélinisante du système nerveux central qui survient de façon aigue chez un enfant bien portant dans un contexte fébrile et associant des troubles de la conscience, des signes de déficit neurologique et des lésions de la substance blanche à l'IRM [1, 2]. L'incidence de l'ADEM est estimée chez l'enfant à 0,4/100 000 par an [3]. L'ADEM est plus fréquente chez l'enfant comparé à l'adulte [4]. Une prépondérance masculine est rapportée dans plusieurs cohortes pédiatriques avec un sexe ratio entre 1,25 et 1,66 [5, 6]. Dans notre étude, il est à 1,25. Cette tendance est moins nette chez l'adulte [3]. Il semble exister une prédominance saisonnière, avec un pic en hiver et un au printemps [3, 6].

Il s'agit d'une maladie auto-immune dont le mécanisme est mal connu, liée très probablement à l'homologie de structure entre un facteur déclenchant (agent infectieux, vaccin) et des antigènes myéliniques de l'hôte [4, 7]. Une infection ou une vaccination sont les facteurs déclenchants les plus fréquents. L'antécédent infectieux est identifié dans la majorité des cas, similaire à ce qui est rapporté dans les séries pédiatriques. On retrouve ceux-ci dans environ 75% des cas de l'enfant [8, 9] et dans 45 à 50% des cas de l'adulte [4, 10] survenant en général après un intervalle libre de 2 à 30 jours [1]. Dans notre série une infection virale prodromique est notée dans 44,5%. La symptomatologie clinique de l'ADEM associe des signes généraux: fièvre, nausées, vomissements, malaise, céphalées... et des manifestations neurologiques: d'encéphalopathie et des signes neurologiques focaux hémisphériques ou médullaires. Les troubles de conscience sont observés dans 19 à 69% des cas d'ADEM [5, 9] associés à des convulsions dans 13 à 35% des cas [9, 11]. La fièvre est observée dans 43 à 52% des cas [4, 5, 11]. Dans notre étude les troubles de conscience et les crises convulsives sont présents dans 55,5% des cas; par contre la fièvre est présente dans 100% des cas avec des fébricules ne dépassant pas 38°C dans 33% des cas. L'IRM cérébrale représente l’élément clé du diagnostic, en objectivant des lésions typiquement multiples et disséminées, prédominant au niveau de la substance blanche. L'atteinte de la substance grise profonde (thalamus, noyaux gris centraux) est peut être notée dans 15 à 60% des cas [4]. Dans notre série l'atteinte de la substance blanche était présente dans 100% des cas, associée à des lésions de la substance grise dans 44,5% des cas.

L’étude du LCR permet d'exclure les diagnostics différentiels telle une méningo-encéphalite infectieuse justifiant un traitement urgent et spécifique. L’étude peut objectiver une anomalie non spécifique à type de pléïocytose lymphocytaire associée à une hyper-protéinorachie [12]. Chez nos patients l'analyse de LCR a objectivé une réaction méningée sans syndrome inflammatoire dans huit cas et était normal dans un cas. Pour les modalités du traitement de l'ADEM, une corticothérapie sous forme de bolus de méthyl-prednisolone à dose de 30 mg/kg par jour à répéter 3 jours de suite avec relais per os par de la prednisolone 1 mg/kg par jour pendant 4 à 6 semaines avec une dégression progressive [7, 5, 9, 13]. Tous nos patients ont bénéficié de ce traitement.

L’évolution sans séquelles de l'ADEM dans plus de 50% [4]. L'amélioration clinique est observée généralement dans l'immédiat dans les heures ou les jours suivant l'administration du traitement [12]. Le risque de récidive de l'ADEM est de 15 à 20% dans plusieurs cohortes. La mortalité de l'ADEM est actuellement inférieure à 5% chez l'enfant et d'environ 8% chez l'adulte [3, 4]. Les séquelles neurologiques les plus fréquentes sont des déficits focaux des membres, une ataxie ou des troubles visuels. Les troubles cognitifs et du comportement sont identifies dans 6 à 50% des patients des séries pédiatriques [7]. Dans notre étude l’évolution est favorable dans 89%; les séquelles neurologiques type ataxie dans un cas; la récidive dans un cas et on a déploré le décès d'une patiente vu le tableau clinique initialement fait de détresse respiratoire.

## Conclusion

L'ADEM est une affection rare du système nerveux central chez l'enfant, caractérisée par une démyélinisation de la substance blanche. Le tableau clinique initial peut mimer un tableau sévère d'infection du SNC avec fièvre. Les signes cliniques associent les signes d'encéphalopathie, crises convulsives et des signes généraux nécessitant parfois l'admission en réanimation. Le diagnostic d'ADEM doit être systématiquement évoqué devant un tableau d'encéphalite aiguë inexpliquée. Les critères diagnostiques sont fondés sur les éléments cliniques et les examens radiologiques notamment l'IRM. Le traitement est basé sur la corticothérapie. L'amélioration clinique est parfois spectaculaire dès l'administration des corticoïdes. Cet élément souligne la nécessité de ne pas méconnaître ce diagnostic et de le poser plus précocement pour une bonne évolution.
